# The Potential Role of Circulating Endothelial Cells and Endothelial Progenitor Cells in the Prediction of Left Ventricular Hypertrophy in Hypertensive Patients

**DOI:** 10.3389/fphys.2019.01005

**Published:** 2019-08-09

**Authors:** Magdalena Budzyń, Bogna Gryszczyńka, Maciej Boruczkowski, Mariusz Kaczmarek, Beata Begier-Krasińska, Angelika Osińska, Alicja Bukowska, Maria Iskra, Magdalena Paulina Kasprzak

**Affiliations:** ^1^Department of General Chemistry, Chair of Chemistry and Clinical Biochemistry, Poznań University of Medical Sciences, Poznań, Poland; ^2^Department of Clinical Immunology, Poznań University of Medical Sciences, Poznań, Poland; ^3^Department of Hypertensiology, Angiology, and Internal Diseases, Poznań University of Medical Sciences, Poznań, Poland; ^4^Medical Analysis Laboratory, Regional Blood Center, Poznań, Poland

**Keywords:** left ventricular hypertrophy, hypertension, circulating endothelial cells, endothelial progenitor cells, endothelial dysfunction, heart

## Abstract

**Background:**

The main aim of present study is to evaluate the potential role of circulating endothelial cells (CECs) and endothelial progenitor cells (CEPCs) – representing specific markers of endothelial damage, in the prediction of left ventricular hypertrophy (LVH) in hypertensive patients categorized into two groups; mild (MH) and resistant hypertension (RH).

**Materials and Methods:**

Thirty patients with MH and 28 subjects with RH were involved in the study. In both groups, patients were divided into an LVH and non-LVH group. The control group included 33 age and sex-matched normotensive volunteers. Physical examination, laboratory tests and echocardiography were conducted.

**Results:**

In both the MH and RH group, patients with as well as without LVH demonstrated a higher number of CECs and a lower ratio of CEPCs/CECs as compared to the healthy control. Multiple linear regression analysis showed a positive association of CEPCs with left ventricular mass (LVM) and left ventricular mass index (LVMI), independently of other confounders.

**Conclusion:**

Our results suggest that endothelial injury observed as an elevated CECs number and its impaired regeneration, reflected by a lowered CEPCs/CECs ratio, precede LVH occurrence and may play a significant role in LVH development regardless of the clinical severity of hypertension. Moreover, independent correlation of CEPCs with echocardiographic (ECG) incidences of LVH suggests their potential use as a screening biomarker to stratify the risk of LVH development.

## Introduction

Left ventricular hypertrophy is one of the most common forms of TOD occurring in hypertensive patients, associated with an increased cardiovascular disease-related morbidity and mortality (Ruilope and Schmieder, 2008; Katholi and Couri, 2011; Okwuosa et al., 2014; Shenasa and Shenasa, 2017). An increase in LV wall stress is the principal mechanical factor in the development of LVH. However, recent studies indicate that some other factors, including those connected with endothelial dysfunction and/or damage, may play a significant role in the development and maintenance of LVH (Yildiz et al., 2003; Sciacqua et al., 2006; Peng et al., 2014; Bartnicki et al., 2016). There is evidence that nitric oxide (NO) released from healthy endothelium prevents cardiac hypertrophy, whereas endothelin, a powerful microvascular constrictor and pro-inflammatory substance released from a dysfunctional endothelium, promotes cardiac hypertrophy (Shah, 2005; Bupha-Intr et al., 2012; Poulikakos et al., 2014; Archer et al., 2017). It may suggest that endothelial dysfunction precedes measurable LVH and that investigating therapies aimed at normalizing endothelial function may limit the development of LVH. The prevalence of LVH in hypertensive patients is very high, especially in those with RH, in which this complication occurs at a frequency ranging from 40% to 55% (Cuspidi et al., 2001, 2010). RH represents an extreme phenotype of hypertension, characterized by a high uncontrolled blood pressure (BP), despite sustained therapy with at least three different classes of antihypertensive medications, including a diuretic agent. Recent studies suggest that high susceptibility of RH patients to left ventricular remodeling may be associated with their worse endothelial status (Figueiredo et al., 2012; Chang-Min et al., 2014).

Due to the close relationship between endothelial abnormalities and LVH in hypertensive patients, a growing number of studies have been focused on the implication of parameters of endothelial dysfunction and/or damage in the diagnosis and management of cardiac hypertrophy (Palmieri et al., 2005; Salles et al., 2007; Yeboah et al., 2010). The presence of CECs has recently been recognized as a direct and specific marker for endothelial dysfunction and/or injury (Goon et al., 2005; Erdbruegger et al., 2006). The mechanisms of detachment of endothelial cells from vessel walls include their mechanical damage and/or apoptosis, proteolysis of subendothelial matrix proteins or a deficiency of anchoring proteins (Scheller et al., 2014). It was found that an elevated number of CECs is associated with an increased cardiovascular risk as well as worsening cardiovascular outcomes (Boos et al., 2006, 2007a, b). A related circulating cell population is CEPCs, which originate from bone marrow (Kawamoto and Asahara, 2007; Asahara et al., 2011; Scheller et al., 2014). CEPC numbers tend to increase in the peripheral blood following vascular injury. The mobilized CEPCs migrate to the sites of injured endothelium and differentiate into mature endothelial cells *in situ* (Dimmeler and Zeiher, 2004; Lee and Poh, 2014). Thus, a balance between the CEPCs and CECs seems to be critical for effective endothelial regeneration, which assures continuity of endothelial lining. Therefore, CEPCs/CECs ratio is treated as a reliable parameter of the body’s capacity for endothelial repair (Karthikeyan et al., 2011; Szpera-Goździewicz et al., 2017).

In our previous work, we demonstrated a higher number of CECs and a drastically lowered CEPCs/CECs ratio in patients with mild (MH) and RH (Budzyń et al., 2018). In the present study, for the first time, we tried to determine the potential of these cells in the prediction of LVH in the same group of hypertensive patients. Therefore, patients were divided into those with and those without LVH, and the level of CECs, CEPCs and their ratio were evaluated and compared to a normotensive control. Moreover, in each group of hypertensive patients, the correlation of CECs, CEPCs and their ratio with echocardiographic (ECG) incidences of LVH were also investigated.

## Materials and Methods

### Patients

The study was performed in accordance with the principles of the Declaration of Helsinki, and the investigational protocol was approved by the Local Bioethical Committee of Poznań University of Medical Sciences (no. 163/17). The study was carried out in a group of hypertensive patients (38 men and 20 women), aged between 21 and 73 (mean age 52.46 ± 11.37) who had been admitted to the Department of Hypertension at the University of Medical Sciences in Poznań. The control group consisted of 33 normotensive blood donors of the Regional Blood Center in Poznań (25 men and eight women), aged between 27 and 61 (mean age: 41.87 ± 6.99), who had no symptoms and/or signs of cardiovascular disease. Written informed consent was obtained from all participants. All patients underwent laboratory and physical examination, including BP measurements performed three times at rest, in a supine position, in standard condition, using a validated upper-arm BP monitor (Omron 705IT). Based on the detailed interview and a clinical examination, the patients were divided into two groups: patients with MH including 20 men and 10 women (mean age 52.87 ± 13.55) and patients with RH comprising 18 men and 10 women (mean age 56.27 ± 10.78). Resistant arterial hypertension was recognized when, despite the use of at least three antihypertensive agents (including a diuretic) in maximum doses, it was impossible to achieve the target values of arterial BP lower than 140/90 mmHg. According to the results of the ECG measurement, hypertensive patients belonging to the MH and RH group, respectively, were divided into LVH and non-LVH. Doppler ultrasound of the renal arteries was performed to exclude secondary causes of arterial hypertension. The exclusion criteria were as follows: secondary hypertension; white coat hypertension; myocardial infarction and revascularization within 6 months before the study; stroke and transient ischemic attack (TIA) within 6 months before the study; congestive heart failure with grade III-IV according to New York Heart Association grading; chronic kidney disease defined when eGFR <30 ml/min per 1.73 m^2^ for >3 months according to the Kidney Foundation’s Kidney National Disease Outcomes Quality Initiative; addiction to alcohol and psychotropic substances, active cancer, diabetes or infections within 6 weeks prior to the study. Demographics and clinical characteristics of study subjects were given in Table 1.

**TABLE 1:** Clinical baseline characteristics of the study subjects.

**Parameter**	**Control (*n* = 33)**	**MH group (*n* = 30)**	**RH group (*n* = 28*)***
Age (years)	41.87 ± 6.99	52.87 ± 13.55	56.27 ± 10.78
Gender F/M	8/25	10/20	10/18
Smoker (n)	3	5	4
SBP (mmHg)	112 ± 7	144 ± 16^*^	172 ± 21^∗∗^
DBP (mmHg)	75 ± 6	84 ± 11^*^	93 ± 11^∗∗^
BMI (kg/m^2^)	25 ± 4	28 ± 5	30 ± 6^∗∗^
WBC (10^9^/L)	6.06 (5.21–7.05)	7.04 (5.68–8.89)^*^	6.95 (5.69–8.60)^∗∗^
NEUT (10^9^/L)	3.08 (2.49–3.71)	4.27 (3.29–5.71)^*^	4.42 (3.55–5.85)^∗∗^
MONO (10^9^/L)	0.57 (0.44–0.67)	0.46 (0.34–0.53)	0.45 (0.28–0.67)
LYMTH (10^9^/L)	2.17 (1.81–2.45)	1.64 (1.39–2.37)^*^	1.82 (1.37–2.15)^∗∗^
RBC (10^12^/L)	4.97 (4.87–5.20)	4.80 (4.44–5.13)	4.60 (4.34–5.07)
PLT (10^9^/L)	228 (200–278)	215 (173–267)	224 (170–272)
HGB (g/dL)	15 ± 1.09	12.5 ± 2.42	14.67 ± 3.21
**Drug intake**			
Antiplatelet agents (n)	0	0	0
Anticoagulants (n)	0	0	0
Thrombolytic drugs (n)	0	0	0
Anti-hypertensive drugs (n)	0	3.5	1.9
β-blockers (n)	0	16	9
Angiotensin-converting enzyme inhibitors (ACE-I) (n)	0	21	15
Angiotensin II receptor antagonists (ARB) (n)	0	9	12
Calcium antagonists (n)	0	25	9
Diuretics (n)	0	30	8
Aldosterone antagonists (n)	0	11	2
Concomitant lipid-lowering therapy – statins (n)	0	24	26
Non-steroidal anti-inflammatory drugs (NSAID) (n)	0	0	0
Proton pump inhibitors (n)	0	11	12

BMI, body mass index; SBP, systolic blood pressure; DBP, diastolic blood pressure; WBC, white blood cells; NEUT, neutrophils; MONO, monocytes; LYMTH, lymphocytes; RBC, red blood cells; PLT, platelets; HGB, hemoglobin. ^*^MH vs. control *p* ≤ 0.05, ^∗∗^RH vs. control *p* ≤ 0.05.

### Echocardiographic Examination

In all MH and RH patients, a complete transthoracic ECG study with Vivid S6 (GE Medical System, Tirat Carmel, Israel) with a 1,5 to 3,6 MHz matrix cardiac sector probe was carried out. Linear measurements were made according to the European Society of Echocardiography. All ECG measurements were performed by a cardiologist with subspecialty training in echocardiography. RWT was defined as: RWT = 2 × LVPWd/LVDd, where: LVPWd – posterior wall diastolic thickness, LVDd (Lang et al., 2005). LVM was calculated according to the Devereux formula: LVM (g) = 0.8 × 1.04 [(IVSd + LVDd + LVPWd)^3^ − LVDd^3^] + 0.6, where: LVDd, IVSd, LVPWd – posterior wall diastolic thickness (Devereux et al., 1986). The LVMI was obtained as an indicator of LVH by echocardiography as a ratio of LVM and body surface area. LVH was defined as: LVMI >125 g/m^2^ for men and >110 g/m^2^ for women.

### Laboratory Analysis

Blood samples were drawn at early morning, from the arms of hypertensive patients, in the recumbent position after 10 min of rest. Blood was collected from each patient into ethylenediaminetetraacetic acid (EDTA) anticoagulant and used for flow cytometric analysis. Multicolor flow cytometry analysis was performed according to the method published by Szpera-Goździewicz et al. (2017) and modified by us elsewhere (Budzyń et al., 2018). The conjugated mouse anti-human monoclonal antibodies: CD34, CD146, CD45, and CD133 (BioLegend, London, United Kingdom) were used. The data were analyzed using BD FACSDiva software. The number of CD45 (−), CD34 (+), CD146 (+), CD133 (−) cells per 1,000,000 analyzed nucleated cells was defined as CECs, whereas CD45(−), CD34 (+), CD146 (+), and CD133 (+) cells as EPCs. The calibration of flow cytometry and the control of fluid stability were conducted each time before analysis. The results were expressed as the number of CECs and/or CEPCs per 4 ml of blood.

### Statistical Analysis

The statistical analysis was conducted using GraphPad Prism software 6.0 (GraphPad Software, San Diego, CA). The normality of quantitative variables was tested using the Kolmogorov*–*Smirnov or Shapiro–Wilk test. Any parameter not following the normal distribution was presented as a median and interquartile ranges and analyzed using non-parametric Mann-Whitney test. Categorical data and proportions were compared using Chi-square or Fisher’s exact test, where appropriate. Normally distributed, continuous variables were presented as a mean and standard deviation and analyzed using the Student’s *t*-test. Multiple group comparisons were performed by one-way analysis of variance or Kruskal–Wallis test, respectively. The Pearson or the Spearman correlation coefficient was used to test the strength of any association between different variables. In all cases, *P*-value ≤0.05 was considered significant.

## Results

### Clinical Characteristic and Echocardiographic Parameters in MH and RH Group

No significant difference was found between the RH and MH group in terms of age, gender, BMI and glucose concentration (Table 2). The RH group demonstrated a higher concentration of cholesterol, hsCRP, urea and an elevated value of SBP as well as DBP compared to the MH group (Table 2). Moreover, a lower eGFR value was observed in RH patients in comparison with the MH group (Table 2).

**TABLE 2:** Comparison of biochemical parameters between MH and RH group.

**Parameter**	**MH (*n* = 30)**	**RH (*n* = 28)**	***p*-value**
Age (years)	53 ± 14	56 ± 10	NS^b^
Gender F/M (*n*)	11/20	12/18	NS^c^
BMI (kg/m^2^)	28 ± 5	30 ± 2	NS^b^
SBP (mmHg)	144 ± 16	172 ± 21	<0.001^b^
DBP (mmHg)	84 ± 11	93 ± 11	0.002^b^
Glucose (mmol/L)	5.60 (5.05–6.41)	5.56 (5.20–6.27)	NS^a^
Cholesterol (mmol/L)	4.04 (2.10–4.86)	5.11 (4.04–5.86)	0.010^a^
Triglyceride (mmol/L)	1.19 (0.95–1.55)	1.09 (0.81–1.62)	NS^a^
HDL-C (mmol/L)	1.62 ± 0.38	1.66 ± 0.54	NS^b^
LDL-C (mmol/L)	2.71 ± 1.47	2.76 ± 0.98	NS^b^
hsCRP (mg/L)	1.60 (0.90–3.55)	3.98 (1.55–8.21)	0.039^a^
Creatinine (μmol/L)	84.2 (65.6–91.6)	82.9 (70.6–111)	NS^a^
Urea (μmol/L)	5.06 (4.15–6.13)	5.62 (5.23–7.25)	0.002^a^
Uric acid (μmol/L)	347 ± 113	343 ± 68	NS^b^
eGFR (ml/min/1.73 m)	86 (76–90)	80 (62–86)	0.040^a^

*^*a*^Results shown as median and interquartile range, comparison done by using Mann–Whitney test. ^*b*^Results shown as mean ± standard deviation, comparison done by using unpaired *t*-test. ^*c*^Categorical data, Fischer’s exact test was used for comparison. BMI, body mass index; SBP, systolic blood pressure; DBP, diastolic blood pressure; HDL-C, HDL cholesterol; LDL-C, LDL cholesterol; hsCRP, highly sensitive C-reactive protein; Cr, creatinine; UA, uric acid; U, urea; eGFR, estimated glomerular filtration rate.*

The ECG parameters of LVH, including LVDd, IVSd, LVPWd, LVM, LVMI, RWT, LVEF are summarized in Table 3. There was no difference in LVEF between the two groups of hypertensive patients. LVM as well as LVMI were significantly higher in the RH group compared to the MH group. Interventricular septum end diastolic diameter (IVSd), left ventricular posterior wall end-diastolic diameter (LVPWd) and RWT were greater in the RH group compared to the MH group. However, LVDd was not significantly different between the two patient groups.

**TABLE 3:** Echocardiographic parameters in MH and RH group.

**Parameter**	**MH (*n* = 30)**	**RH (*n* = 28)**	***p*-value**
LVDd (mm)	47 ± 6	48 ± 5	NS^b^
IVSd (mm)	12 ± 1	13 ± 4	0.009^b^
LVPWd (mm)	11 ± 1	12 ± 9	<0.0001^b^
LVM (g)	211 ± 51	256 ± 62	0.007^b^
LVMI (g/m^2^)	100 ± 23	123 ± 26	<0.001^b^
RWT	0.46 ± 0.06	0.53 ± 0.23	0.002^b^
LVEF (%)	60 (55–65)	65 (60–63)	NS^a^

*^*a*^Results shown as median and interquartile range, comparison done by using Mann-Whitney test. ^*b*^results shown as mean ± standard deviation, comparison done by using unpaired *t-*test. LVDd, left ventricular diastolic diameter; IVSd, interventricular septum diastolic diameter; LVPWd, left ventricular posterior wall diastolic diameter; LVM, left ventricular mass; LVMI, left ventricular mass indexed to body surface; RWT, relative wall thickness; LVEF, left ventricular ejection fraction.*

### CECs, CEPCs and Their Ratio (CEPCs/CECs) in Hypertensive Patients With and Without LVH

**FIGURE 1:**
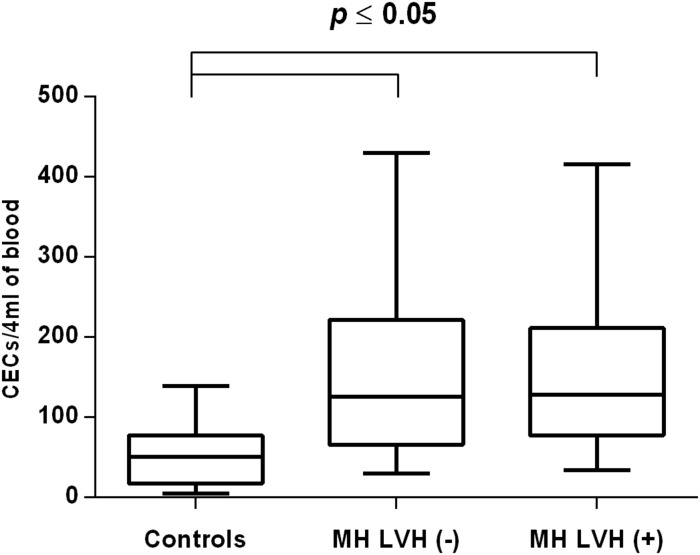
CECs number in patients with and without LVH belonging to MH group. Box and whisker plots show median (central line), upper and lower quartiles (box) and range excluding outliers (whiskers). Data were analyzed using Kruskal–Wallis test followed by the Dunn’s multiple comparison test. *P* ≤ 0.05 was considered statistically significant.

**FIGURE 2:**
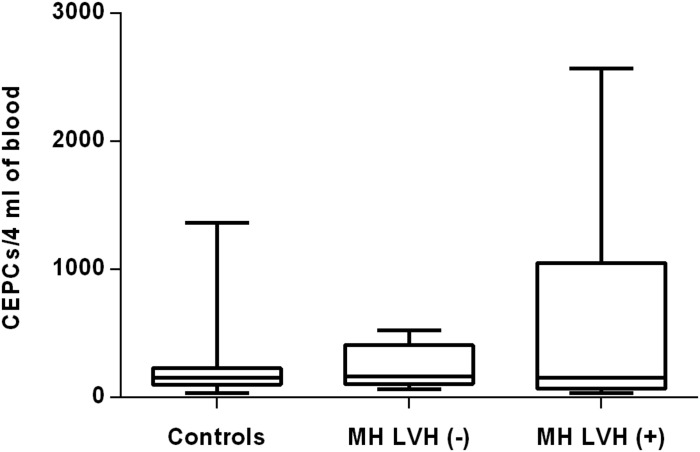
CEPCs number in patients with and without LVH belonging to MH group. Box and whisker plots show median (central line), upper and lower quartiles (box) and range excluding outliers (whiskers). Data were analyzed using Kruskal-Wallis test followed by the Dunn’s multiple comparison test. *P* ≤ 0.05 was considered statistically significant.

**FIGURE 3:**
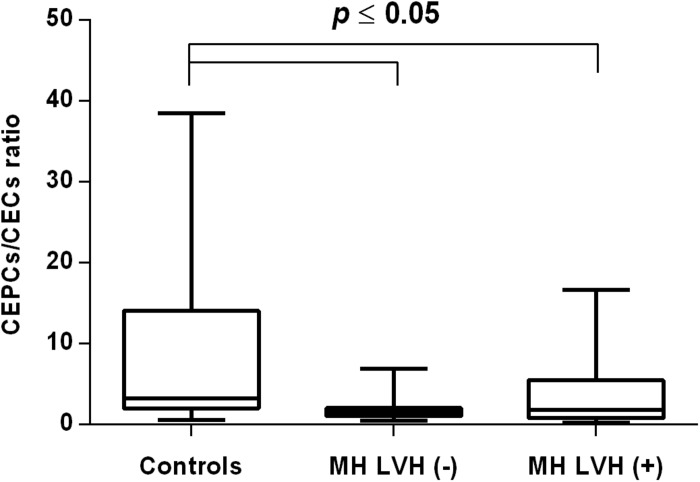
CEPCs/CECs ratio in patients with and without LVH belonging to MH group. Box and whisker plots show median (central line), upper and lower quartiles (box) and range excluding outliers (whiskers). Data were analyzed using Kruskal-Wallis test followed by the Dunn’s multiple comparison test. *P* ≤ 0.05 was considered statistically significant.

**FIGURE 4:**
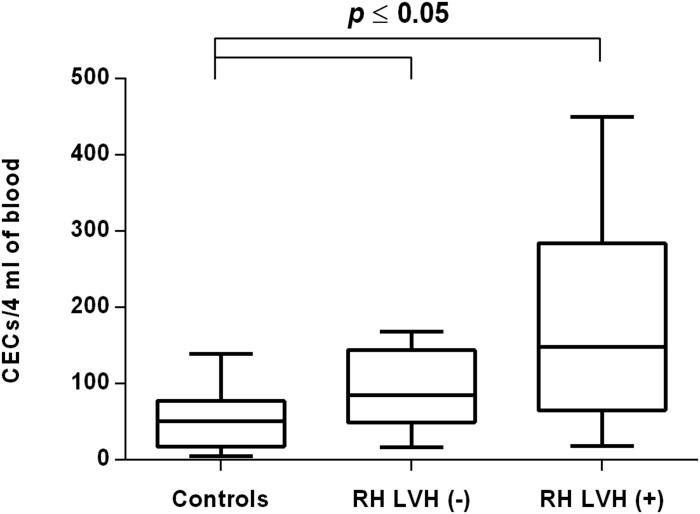
CECs number in patients with and without LVH belonging to RH group. Box and whisker plots show median (central line), upper and lower quartiles (box) and range excluding outliers (whiskers). Data were analyzed using Kruskal-Wallis test followed by the Dunn’s multiple comparison test. *P* ≤ 0.05 was considered statistically significant.

**FIGURE 5:**
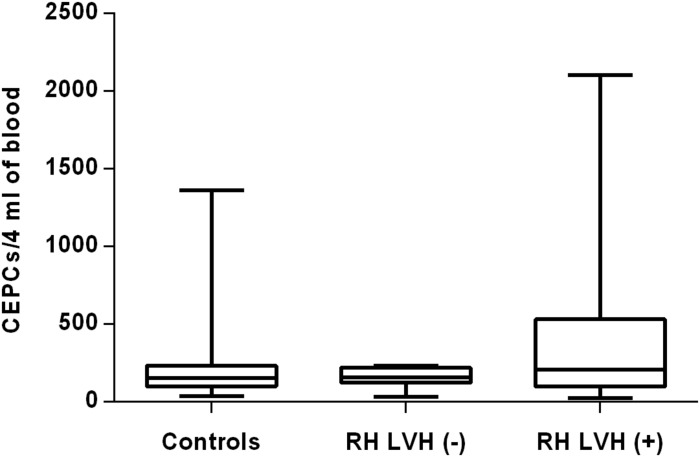
CEPCs number in patients with and without LVH belonging to RH group. Box and whisker plots show median (central line), upper and lower quartiles (box) and range excluding outliers (whiskers). Data were analyzed using Kruskal-Wallis test followed by the Dunn’s multiple comparison test. *P* ≤ 0.05 was considered statistically significant.

**FIGURE 6:**
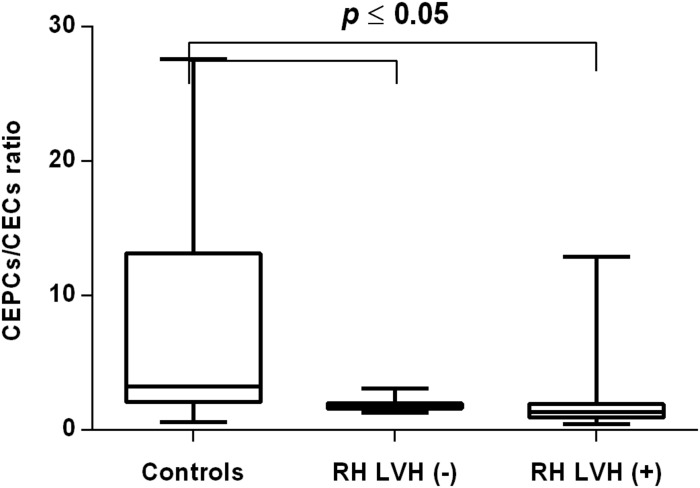
CEPCs/CECs ratio in patients with and without LVH belonging to RH group. Box and whisker plots show median (central line), upper and lower quartiles (box) and range excluding outliers (whiskers). Data were analyzed using Kruskal-Wallis test followed by the Dunn’s multiple comparison test. *P* ≤ 0.05 was considered statistically significant.

In both groups, patients were divided into those with and those without LVH and analyzed parameters of endothelial dysfunction were compared with normotensive control. Among the MH group, patients with as well as without LVH demonstrated a higher number of CECs [128 (77–211); 126 (66–222) vs. 50 (17–77), *P* < 0.0001] and a lower ratio of CEPCs/CECs [1.83(0.81–5.51); 1.55(1.04–2.06) vs. 3.24 (2.03–14), *P* < 0.0001] (Figures 1, 3). However, no statistical difference in the number of CEPCs in MH patients with and without LVH, in comparison with control group was stated [153(67–1051); 167(106–408) vs. 153 (102–232), *P* = 0.609] (Figure 2). The same results were observed in RH patients with and without LVH. They both demonstrated an elevated number of CECs [143 (65–282); 84 (49–142) vs. 50 (17–77), *P* < 0.0001] and a lower ratio of CEPC/CECs [1.32 (0.94–1.97); 1.73 (1.60–2.1) vs. 3.24 (2.03–14), *P* = 0.001] (Figures 4, 6), whereas the CEPC number remained the same in comparison with normotensive subjects [206 (99–536); 155 (123–224) vs. 153 (102–232), *P* = 0.302] (Figure 5).

### CECs, CEPCs and Their Ratio (CEPCs/CECs) in Hypertensive Men With and Without LVH

To exclude the potential effect of gender, especially the influence of menopause status in women on analyzing endothelial parameters, in the second part of research only hypertensive men divided into appropriate groups were taken into consideration. Among the MH as well as the RH group of hypertensive men, a higher number of CECs was demonstrated in those with and without LVH in comparison with men of the control group (Table 4). Moreover, in RH men with LVH the number of CECs was higher in comparison with RH men without LVH (Table 4). In MH men, the number of CEPCs remained the same in those with as well as those without LVH in comparison with normotensive subjects (Table 4). However, in the RH group the number of CEPCs was significantly higher in men with LVH in comparison with the control (Table 4). In the MH as well as the RH group a lower ratio of CEPC/CECs was observed in men with, as well as without LVH (Table 4).

**TABLE 4:** CEPCs, CECs and their ratio in hypertensive men with and without LVH.

	**MH ♂**		**Control ♂**
**Parameter**	**LVH (−)**	**LVH (+)**	
CECs/4 ml	140 (65–251)	107 (40–155)	44 (13–75)^*^
CEPCs/4 ml	167 (106–406)	130 (88–1296)	119 (75–220)
CEPCs/CECs	1.55 (1.02–1.86)	2.25 (1.47–10.82)	3.24 (2.09–13.14)^*^

	**RH ♂**		**Control ♂**
**Parameter**	**LVH (−)**	**LVH (+)**	

CECs/4 ml	102 (68–168)	223 (110–321)^∗∗^	44 (13–75)^*^
CEPCs/4 ml	164 (123–221)	280 (129–629)	119 (75–220)^∗∗∗^
CEPCs/CECs	1.60 (1.31–1.81)	1.33 (0.82–2.79)	3.24 (2.09–13.14)^*^

Results shown as median and interquartile range, comparison done by using Kruskal-Wallis test followed by the Dunn’s multiple comparison test ^*^LVH(−), LVH(+) vs. Control *p* ≤ 0.05, ^∗∗^LVH(+) vs. LVH(−) *p* ≤ 0.05, ^∗∗∗^ LVH(+) vs. Control *p* ≤ 0.05.

### The Association of CECs, CEPCs and Their Ratio With Left Ventricular Hypertrophy Parameters in MH and RH Group

In the first step, univariate analysis was performed to determine the association between LVH parameters and the markers of endothelial injury in each group of hypertensive patients separately. In the next step, multivariate linear regression analysis was performed in a whole group of hypertensive patients to determine the endothelial injury as an independent factor potentially influencing LVH measured by the LVM and LVMI value.

A univariate analysis revealed that CEPCs/CECs correlated positively with LVEDd and negatively with RWT in the MH group (Table 5). In RH the CEPCs number as well as its ratio with CECs correlated positively with IVSd, LVPWd and LVMI (Table 5). Moreover, in RH a significant positive correlation between CEPCs and LVM was found (Table 5).

**TABLE 5:** Univariate analysis of relationship between left ventricular hypertrophy parameters and markers of endothelial injury in MH and RH group.

**MH**			
**Variables**	**CECs**	**CEPCs**	**CEPCs/CECs**

LVEDd (mm)	–0.222	0.080	0.450^*^
IVSd (mm)	–0.063	–0.289	–0.223
LVPWd (mm)	–0.078	–0.158	–0.080
LVM (g)	–0.186	–0.098	0.185
LVMI (g/m^2^)	–0.104	–0.019	0.205
RWT	0.130	–0.196	−0.431^*^

**RH**			

**Variables**	**CECs**	**CEPCs**	**CEPCs/CECs**

LVDd (mm)	0.329	0.049	–0.102
IVSd (mm)	0.010	0.506^∗∗^	0.382^*^
LVPWd (mm)	0.098	0.524^∗∗^	0.449^*^
LVM (g)	0.201	0.529^∗∗^	0.332
LVMI (g/m^2^)	0.085	0.578^∗∗^	0.462^*^
RWT	–0.201	0.323	0.329

Results shown as Spearman’s rank correlation coefficient, ^*^p ≤ 0.05, ^∗∗^*p* ≤ 0.01. LVEDd, left ventricular diastolic diameter; IVSd, interventricular septum diastolic diameter; LVPWd, left ventricular posterior wall diastolic diameter; LVM, left ventricular mass; LVMI, left ventricular mass indexed to body surface; RWT, relative wall thickness.

In a multivariate analysis, taking into consideration both groups of hypertensive patients, only the CEPCs number was associated with an increased value of LVM and LVMI, independently of other factors considered, including the type of hypertension, SBP and DBP (model 2); age, sex and BMI (model 3); hsCRP, eGFR and cholesterol concentration (model 4) (Table 6).

**TABLE 6:** Multivariate analysis of relationship between indicators of left ventricular function and endothelial parameters.

	**LVM**		**LVMI**	
	**Coefficient**	***P-*value**	**Coefficient**	***P-*value**
**Model used**				
**Model 1**				
CECs/4 ml	–0.052	0.406	–0.021	0.645
CEPCs/4 ml	0.041	0.025^*^	0.018	0.024^*^
CEPCs/CECs ratio	–0.150	0.643	–0.100	0.573
**Model 2**				
CECs/4 ml	–0.054	0.472	–0.022	0.532
CEPCs/4 ml	0.042	0.029^*^	0.018	0.032^*^
CEPCs/CECs ratio	–0.068	0.792	–0.029	0.835
**Model 3**				
CECs/4 ml	–0.053	0.523	–0.011	0.672
CEPCs/4 ml	0.036	0.028^*^	0.020	0.026^*^
CEPCs/CECs ratio	–0.191	0.568	–0.108	0.416
**Model 4**				
CECs/4 ml	–0.105	0.237	–0.056	0.179
CEPCs/4 ml	0.042	0.049^*^	0.020	0.043^*^
CEPCs/CECs ratio	–0.076	0.210	–0.046	0.784

Model 1: unadjusted model; model 2 adjusted for type of hypertension, SBP and DBP; model 3 adjusted for age sex and BMI; model 4 adjusted for hsCRP, CHOL and eGFR. ^*^Statistically significant.

## Discussion

The cardiac manifestations of systemic hypertension include LVH at early stages and dilatation with left ventricular dysfunction at late decompensated stages (Gatzoulis et al., 2000; Kahan and Bergfeldt, 2005; Cuspidi et al., 2012). Both have been associated with an increased incidence of ventricular arrhythmias and sudden cardiac death (Tin et al., 2002; Shenasa and Shenasa, 2017). LVH is an adjustment mechanism to chronic pressure overload in arterial hypertension, which allows the left ventricle to maintain volume output against the increased systolic pressure. The hypertrophy is a risk factor for morbidity and mortality in hypertension – the presence of LVH worsens the prognosis in hypertensive patients (Hawkins et al., 2007; Pavlíček et al., 2016). It means that an earlier recognition as well as an improved understanding of cardiac hypertrophy may lead to more effective therapeutic strategies for this cardiovascular risk factor.

Echocardiography is now frequently used in the evaluation of LVH in the hypertensive patients (Zheng et al., 2010; Pavlíček et al., 2016). It has proven to be a reliable and reproducible technique in the assessment of cardiac anatomy and function. Assessing left ventricular mass (LVM) and/or LVM indexed by body surface area (LVMI) is the most common role of echocardiography in hypertensive patients. Recent studies indicate that endothelial dysfunction is linked with LVH parameters in the general population independently of other cardiovascular risk factors (Lazdam et al., 2012), and there is evidence that a declining endothelial function may identify an adverse cardiac phenotype in healthy young adults (Yeboah et al., 2010).

Nitric oxide plays the central role in endothelial function. Recent experimental studies have firmly established that NO released from endothelial cells exerts several specific effects on myocardial function, analogous to the endothelial regulation of vascular wall function (MacCarthy and Shah, 2000). In particular, these include the selective enhancement of myocardial relaxation, diastolic LV function and reduction in myocardial O_2_ consumption (Rastaldo et al., 2007; Ruiz-Hurtado and Delgado, 2010). Moreover, it must considered that endothelial cells secrete other vasodilation and vasoconstrictor factors which modulate cardiac performance, and their imbalance contributes to pathological cardiac remodeling (Segers et al., 2018). Accordingly, there is little doubt that endothelial damage and/or dysfunction has a larger impact on cardiac function and heart failure progression than currently anticipated (Segers et al., 2018). In this context, parameters of endothelial dysfunction can be recognized as potential diagnostic and prognostic biomarkers of the cardiac remodeling process in hypertensive individuals, even though there is not quite enough evidence from large clinical trials.

Only a few studies demonstrate a relationship between endothelial dysfunction and LVH in hypertensive patients. An increased concentration of biochemical markers of endothelial dysfunction, such as inter-cellular adhesion molecule 1 (ICAM-1), vascular cell adhesion protein 1 (VCAM-1) and E-selectin, were observed in hypertensive patients with LVH (Kuroda et al., 2001; Malmqvist et al., 2002). Moreover, endothelial damage reflected by abnormal albuminuria was independently associated with LVH occurrence in hypertensive patients, including those with RH (Plavnik et al., 2002; Salles et al., 2007; Nabbaale et al., 2015). However, all of the previously analyzed parameters of endothelial dysfunction are not highly specific and their level is subject to intra-patient variability and also strongly depends on renal function. Therefore, the main aim of our study is to examine the association of specific circulating markers of endothelial damage: CECs and CEPCs with ECG parameters of LVH in MH and RH, the latter being a well-known predisposing factor for cardiac hypertrophy development. We observed that a higher number of CECs reflecting endothelial damage already occurs in patients without LVH and keep rising in patients with LVH, in both the MH and RH group. Ercan et al. (2003) obtained results similar to ours, i.e., a worse endothelial function assessed by FMD in hypertensive patients with and without LVH in comparison to the control group. These observations suggest that endothelial injury may occur prior to LVH development and contribute significantly to cardiac remodeling. Moreover, our studies indicate that the participation of endothelial dysfunction in cardiac hypertrophy may be a mechanism that commonly occurs in various clinical types of hypertension. The important question is whether endothelial perturbation is restricted to coronary circulation or rather spreads out in the peripheral circulatory bed. There are findings indicating that patients with non-ischemic heart failure including LVH tend to have coronary rather than systemic endothelial dysfunction (Canetti et al., 2003; Dini et al., 2009; Shantsila et al., 2012). It has been proven that coronary endothelial impairment is strictly connected with reduced NO synthase expression and activity in cardiac tissue, consequently leading to the deterioration of cardiac contractility, cardiac remodeling and fibrosis (Lapu-Bula and Ofili, 2007). However, in our study an elevated number of CECs was detected in peripheral blood samples suggesting the systemic nature of endothelial dysfunction in hypertensive patients including those with LVH. Our results are in accordance with previous observations made by other authors who detected peripheral endothelial dysfunction in ischemic as well as non-ischemic heart failure (Kubo et al., 1991; Bitar et al., 2006). Interesting results have been obtained by Heitzer et al. (2005) who demonstrated that a lower vasodilator response to acetylcholine and to sodium nitroprusside, that reflected peripheral endothelial dysfunction, is an early manifestation in the progression of left ventricular dysfunction and may even precede the symptoms of heart failure. It seems that the exact localization of the site of endothelial dysfunction contributing to LVH may be difficult mainly due to the complexity of interactions between different segments of the vascular tree. The factors liberated by dysfunctional endothelium in some specific areas of circulation may affect endothelium localized elsewhere. Therefore, in further studies, the concept of endothelium as one organ should be established as the most appropriate.

In response to endothelial damage, the amount of CEPCs should rise adequately. Although in our study the number of CEPCs demonstrated a tendency to rise in hypertensive patients with LVH, it did not reach statistical significance. Similar results were obtained by other authors, who did not observe any statistical difference in the total number of CEPCs in patients with essential hypertension (Delva et al., 2007; Marketou et al., 2014). However, they assessed CEPC levels in the general population of hypertensive patients, not considering their classification based on the absence/presence of LVH. Other authors, who divided hypertensive patients using LVH criteria observed even lowered levels of CEPCs in those with ECG evidence of LVH in comparison to those who did not have LVH (Lee et al., 2011). To find an explanation for this phenomenon it must be taken into consideration that the amount of CEPCs is regulated by two different mechanisms: release of CEPCs from bone morrow and the survival and life span of CEPCs. The various factors common to hypertensive milieu, such as angiotensin II, aldosterone, catecholamines and cytokines liberated by injury tissues are suspected to activate bone morrow stem cells, which undergo differentiation and mobilization (Montaniel and Harrison, 2016). That mechanism explains an elevated number of bone morrow derived immune cells in hypertensive patients which infiltrate peripheral organs causing their dysfunction (Wenzel et al., 2011; Youn et al., 2013; Santisteban et al., 2015). According to a previously described concept, CEPCs which originate from bone morrow probably undergo an analogous process: enhanced activation and mobilization which should result in their rise in the circulation. However, in the circulation the amount of CEPCs is regulated by other mechanisms connected with their lifespan and death, such as apoptosis. Matsumoto et al. who observed an increased activity of caspase-3 in CEPCs isolated from patients with aortic valves stenosis, has indicated that enhanced apoptosis is a significant cause of the reduced CEPCs level (Matsumoto et al., 2009). It means that the enhanced mobilization of CEPCs from bone morrow may be masked by their enhanced elimination from circulation due to apoptosis. Therefore, as a consequence, the overall number of CEPCs observed in circulation do not rise apparently or may even fall despite their continuous liberation from bone morrow. These speculations could be supported by the evaluation of the apoptotic status of CEPCs in hypertensive individuals, which will be our aim in further studies.

Described mechanisms may also explain the positive relationship between CEPCs and ECG parameters of LVH: LVM as well as LVMI was found in RH patients and confirmed by other authors (Oliveras et al., 2008; Kalyva et al., 2016). This correlation may indicate mobilization of CEPCs from bone morrow rising adequately to organ damage, despite the fact that CEPCs level in the circulation do not elevate proportionally, due to failure mechanisms described above. Interestingly, CEPCs remained an independent predictor of LVH in the general population of hypertensive individuals even after the adjustment for other confounders, such as the type of hypertension, SBP and DBP, age and sex, BMI, inflammation, and renal function parameters. It means that LVH progression may be directly associated with an increased liberation of CEPCs from bone morrow. However, considering other described mechanisms controlling CEPCs turnover, the final number of CEPCs seems to be inadequate for endothelial regeneration and organ repair. It is reflected by a lowered CEPCs/CECs ratio which already occurs in hypertensive individuals without LVH and falls further in those with LVH.

Some studies have suggested that CEPC numbers may be affected by age and gender (Fadini et al., 2008; Pelliccia et al., 2009; Rousseau et al., 2010). This hypothesis is derived from observations indicating that the incidence of cardiovascular diseases is lower in cycling women, but after the age of between 45 and 54 y it drastically increases, reaching that of age-matched men within 10 y after menopause (Rousseau et al., 2010). This phenomenon is probably associated with the protective effect of endogenous estrogens on the vasculature. The impact of estrogens on endothelium, including CEPCs level and function, has emerged as a possible mechanism for this phenomenon (Masuda et al., 2007; Fadini et al., 2008; Li et al., 2013). Although estrogens appear to increase bone marrow−derived CEPCs production, reports on sex differences in CEPC levels have been studied in small populations with less consistent findings. Stauffer et al. (2008) found no differences in CEPCs in 29 menopausal women compared with men. Similarly, no sex-related differences in healthy young men and women were reported by Ruszkowska-Ciastek et al. (2015). In contrast, some authors indicate that premenopausal women had a higher CEPCs number compared with menopausal women and age-matched men (Fadini et al., 2008; Lemieux et al., 2009). Most women who qualified for our study were aged above 51 y which corresponds to the average age of menopause occurrence. To minimize the influence of the women participating in our research, we additionally decided to examine all parameters only in the group of men with the exclusion of women. Similarly, as in the general group of hypertensive patients, the CECs were higher in men with and without LVH compared with men in the control group, regardless of the type of hypertension. However, among men with RH, the greatest elevation of CECs, indicating the most severe endothelial damage, occurred in those with LVH complication. Interestingly, in the same group of RH men, a significant elevation of CEPCs was observed, which may be a response to the endothelial injury. These observations, obtained in hypertensive men, differ from those obtained in the general group of patients, including men and women, and probably confirm some gender differences in the endothelial function reported previously (Routledge et al., 2012; Stanhewicz et al., 2018). A growing number of studies suggest that the risk factors and mechanisms of endothelial injury may be strongly sex-dependent and should be analyzed in a population of men and women separately (Routledge et al., 2012; Cattaneo et al., 2017). Some authors speculate that the identification of sex specific mechanisms of endothelial dysfunction may improve patient outcomes across a wide variety of cardiovascular diseases (Bacon et al., 2011). In the present work, the number of patients was too small for the examination of gender variation in the analyzed endothelial parameters, to produce reliable results. However, future clinical studies, concerning the role of endothelium in hypertension, should be considered from a gender perspective.

Admittedly, the assumption about the important role of endothelial injury in LVH development is not innovative and was reported previously by other authors, however, our observations, that this process is accompanied by the disruption in endothelial regeneration before LVH occurrence, are crucial and may have some important clinical implications. They offer insight into the possible biological mechanism underlying LVH pathology and, in this way, enhance the chances of developing an effective targeted therapy for its treatment. A recovery of a sufficient number of functional CEPCs able to regenerate damaged endothelium may have beneficial effects; it may prevent hypertension related complications, including LVH, but may also lead to the regression of hypertension itself.

We are aware of the limitations of our study. It can be agreed that the number of participants was small. Another limitation was the lack of assessment of functional capacity of CEPCs. It is very likely that apart from their level in circulation, this subpopulation of endothelial cells may have an altered functional characteristic, which has a potential impact on cardiac hypertrophy development in hypertensive individuals. Despite these limitations, an important finding derived from our study is the fact that CEPCs may potentially serve as a screening biomarker in hypertensive patients to stratify the risk of LVH and initiate preventive interventions. However, larger clinical studies are necessary to determine the optimal diagnostic threshold values of CEPCs in differentiating hypertensive patients with a high risk of LVH development. It must also be considered that due to the deficiencies in our understanding of CEPCs pathophysiology, these endothelial cells are far from being a serious contender as a clinically useful biomarker. Thus, further experimental research is required to clarify the mechanism underlying the involvement of CEPCs in the pathogenesis of LVH to verify their predictive values.

## Conclusion

In conclusion, our results suggest that endothelial injury, observed as an increased CECs number, and its impaired regeneration, reflected by a lowered CEPCs/CECs ratio precede LVH occurrence and may have a significant role in LVH development, regardless of the clinical severity of hypertension. Moreover, our data shows that CEPCs number is independently associated with LVH in hypertensive individuals, even after the adjustment of some potential confounders. Thus, CEPCs could potentially serve as a screening biomarker in hypertensive patients to stratify risk of LVH and initiate preventive interventions. However, more research is needed to delineate the pathophysiological effects of CEPCs on LVH and to verify their predictive values.

## Ethics Statement

The studies involving human participants were reviewed and approved by the Local Bioethical Committee of Poznań University of Medical Sciences (No. 163/17). The patients and participants provided their written informed consent to participate in this study.

## Author Contributions

MagB contributed to the design of the study and drafted the manuscript. MagB, BG, MacB, MarK, BB-K, AO, AB, MI, and MagK contributed to the conception of the study. MagB, BG, BB-K, AO, and MI contributed to the data acquisition. MagB, MacB, MarK, BB-K, AO, AB, and MagK contributed to the analysis of the study. All authors critically revised the manuscript, gave final approval, and agreed to be accountable for all aspects of work ensuring integrity and accuracy.

## Conflict of Interest Statement

The authors declare that the research was conducted in the absence of any commercial or financial relationships that could be construed as a potential conflict of interest.

## Abbreviations

BMI: body mass index
CECs: circulating endothelial cells
CEPCs: circulating endothelial progenitor cells
DBP: diastolic blood pressure
eGFR: estimated glomerular filtration rate
FMD: flow mediated dilation
HDL-C: HDL-cholesterol
HGB: hemoglobin
hsCRP: highly sensitive C-reactive protein
IL-6: interleukin 6
IVSd: intraventricular septal diameter
LDL-C: LDL-cholesterol
LVDd: left ventricular diastolic diameter
LVEF: left ventricular ejection fraction
LVH: left ventricular hypertrophy
LVMI: left ventricular mass index
LVPWd: thickness of the left ventricular posterior wall
LYMPH: lymphocytes
MH: mild hypertension
MONO: monocytes
NEUT: neutrophils
PLT: blood platelet
RBC: red blood cells
RH: resistant hypertension
RWT: relative wall thickness
SBP: systolic blood pressure
TOD: target organ damage
